# Supplementary material for: Spec2Vec: Improved mass spectral similarity scoring through learning of structural relationships
Source: PLoS Comput Biol. 2021 Feb 16;17(2):e1008724. doi: 10.1371/journal.pcbi.1008724 (PMC7909622; doi:10.1371/journal.pcbi.1008724)
Supplement: S1 Text — (PDF) [file pcbi.1008724.s001.pdf]

# Supporting Information S1 – Data processing and parameter searches

For main article "Spec2Vec: Improved mass spectral similarity scoring through learning of structural relationships", *Florian Huber, Lars Ridder, Stefan Verhoeven, Jurriaan H. Spaaks, Faruk Diblen, Simon Rogers, Justin J.J. van der Hooft.*

Is this supplemental material we present additional details on the data processing workflow and the parameters searches on which parameter choices for the used scores (cosine score, modified cosine score and Spec2Vec) were based.

## Contents of supporting information S1


Data processing protocol ........ 2

Spec2Vec model parameters ........ 3

Parameter tuning for the cosines score ........ 4

Parameter tuning for the modified cosines score ........ 5

# Data processing protocol

**Data pre-processing:**

1. Import all spectra from GNPS website via matchms json import (all spectra file from https://gnps-external.ucsd.edu/gnpslibrary/ALL_GNPS.json from 2020-05-11)
   Found non-empy spectra: 154820 spectra
2. Run default matchms filters to clean, correct, and infer missing metadata. Results in:
   94462 spectra with InChI (15822 unique)
   94155 spectra with Smiles (20542 unique)
   94121 spectra with InChIKey (13505 unique in first 14 characters)
3. Run extensive automated PubChem lookup (code provided in manuscript repository).
   Results in:
   128103 spectra with InChI (17620 unique)
   128052 spectra with Smiles (23097 unique)
   128042 spectra with InChIKey (14978 unique in first 14 characters)

**Creation of subsets:**

Creation of 4 different datasets:

- **AllGNPS:** All spectra after matchms cleaning and PubChem lookups (154820 spectra)
- **AllPositive:** all spectra from AllGNPS dataset with positive ionization mode with at least 10 peaks.
  Total: 95,320 spectra,
  77135 spectra with InChI (14550 unique)
  76996 spectra with Smiles (18690 unique)
  77092 spectra with InChIKey (12847 unique in first 14 characters)
- **AllPositiveAnnotated**
- **UniqueInchiKeys:** Reduced dataset used for benchmarking. Keep only one spectrum for every unique InChIKey from the AllPositive dataset. Spectra are selected by:
  i) If possible, select spectra with > 10 peaks above 1% of maximum peak intensity
  ii) Out of those (if multiple): select best library quality level: 1> 2> 3
  iii) Out of those (if multiple): select spectrum with most peaks above 1% of maximum peak intensity
  iv) if still multiple, pick random!
  This gives 13717 spectra, out of which 12797 spectra contain >= 10 peaks.

**Data post-processing for "classical" similarity scores (cosine, modified cosine)**

1. Remove peaks with m/z outside [0, 1000]
2. Remove spectra with < 10 peaks remaining
3. Remove peaks with intensities < 0.01 maximum peak intensity (smaller peaks will slow down score calculation, but won't contribute much to the overall scores)

**Data post-processing for spec2vec**

1. Remove peaks with m/z outside [0, 1000]
2. Remove spectra with < 10 peaks remaining
3. Reduce number of peaks using matchms "reduce_number_of_peaks" filter. Parameters: n_required = 10, ratio_desired = 0.5
   Spec2vec is comparing spectrum documents using language model analogies. For the underlying word2vec models we aimed at training on documents of roughly comparable size to ensure that spectra will also get comparable attention during model training. The raw data contained spectra with numbers of peaks ranging from 10 (our own set threshold to include spectra) up to several 10,000s of peaks. We hence removed excessive amounts of low intensity peaks. To account for the fact that larger molecules on average show more fragmentation peaks, the maximum number of kept peaks per spectrum was set to scale linearly with the estimated parent mass:

$$max(n_{peaks}) = 0.5 \cdot parentmass.$$

# Spec2Vec model parameters

The underlying word2vec models were trained using the spec2vec Python library running Gensim. The key model parameters were

- Window size = 500
- Word vector dimension = 300
- Negative sampling
- CBOW mode
- Initial learning rate = 0.025
- Learning rate decay per iteration (per epoch) = 0.00025

We compared models after different iterations using the benchmarking dataset **UniqueInchikey** to find suitable numbers of training iterations (fig. S8). Generally training was comparably stable over a larger range of iterations. It converges to stable results when switching off negative sampling, but negative sampling resulted in better overall results (not shown here).

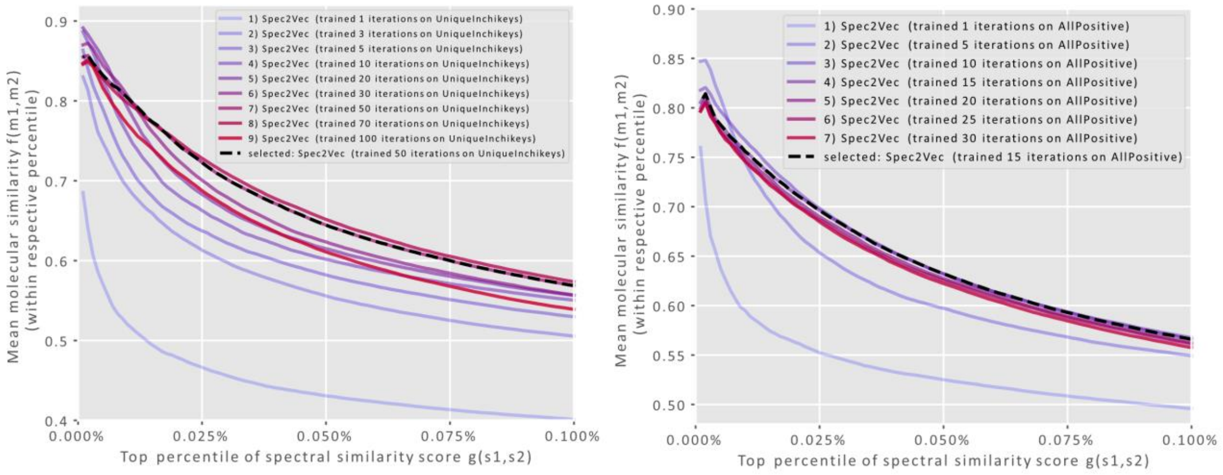


**Fig A.** Word2Vec models were trained on both the **UniqueInchikey** and the **AllPositive** dataset over many iterations. The overall performance was monitored by the correlation between Spec2Vec similarities and structural similarity and revealed that the model rapidly improved during the first 10-15 epochs (iterations). For the **UniqueInchikey** dataset we found the best performance around 50 iterations, for the AllPositive dataset we observed that the changes after 15 iterations were rather minor.

# Parameter tuning for the cosines score

Cosine scores were calculated for all-vs-all spectra of the **UniqueInchikey** dataset using different tolerances and minimum matching peak thresholds.

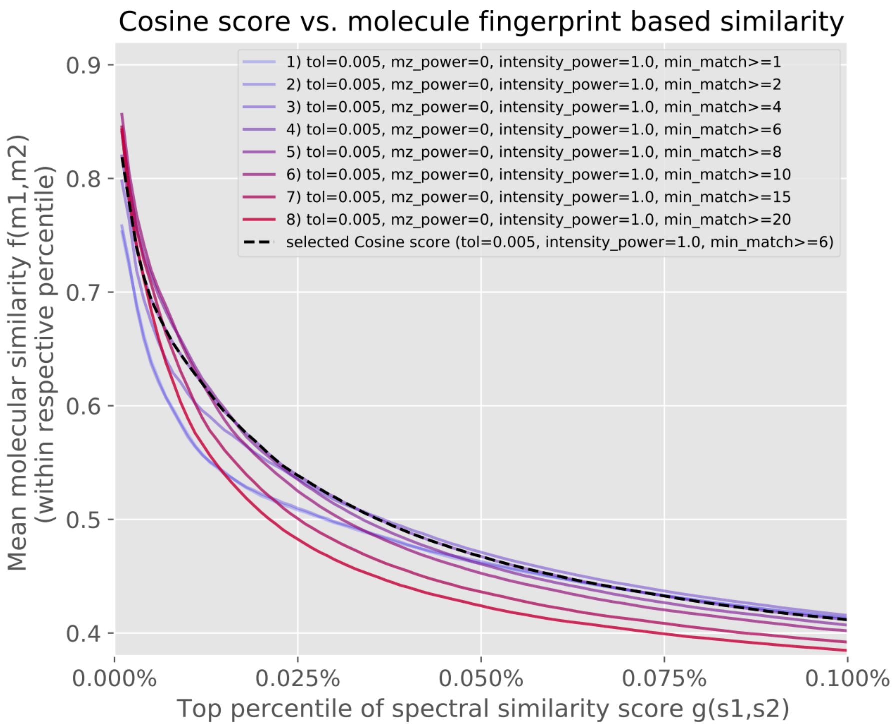


**Fig B**. Parameter search for cosine similarity score. Varying from 1 to 20 the minimum number of matching peaks necessary to calculate a cosine score. For all following comparisons, min_match=6 was chosen.

## Parameter tuning for the modified cosines score

As for the cosine score, modified cosine scores were calculated for all-vs-all spectra of the **UniqueInchikeys** dataset using a tolerance of 0.005 Da together with different minimum matching peak thresholds (Fig C).

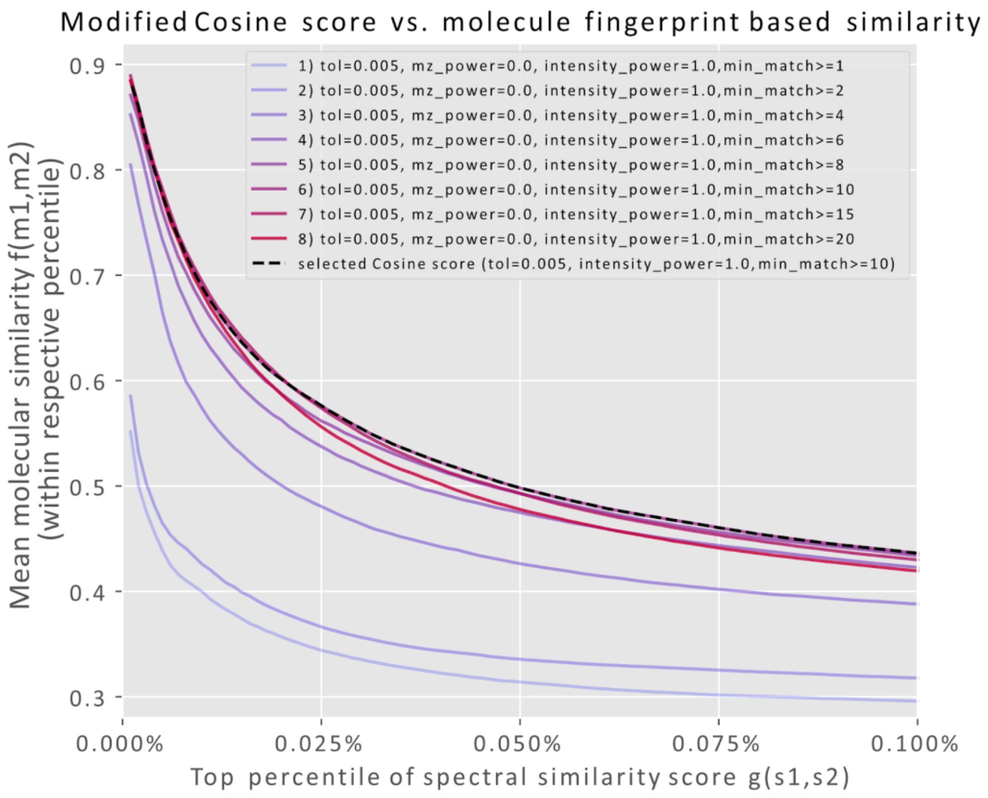


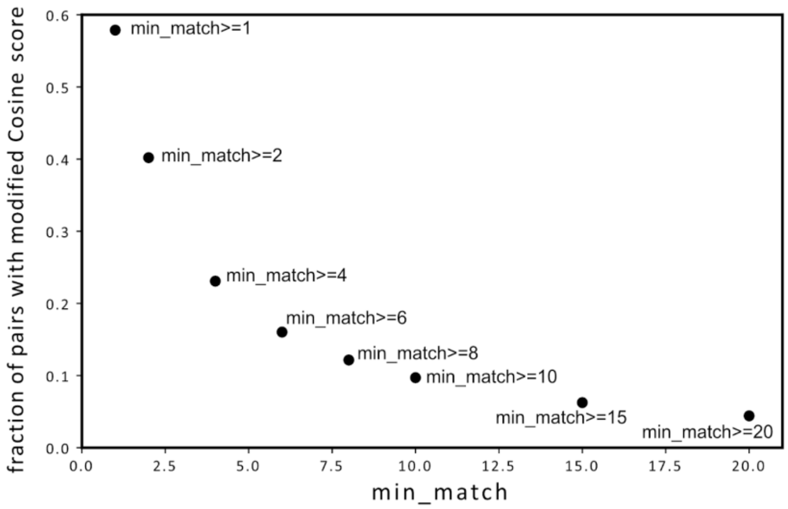


**Fig C**. Parameter search for modified cosine similarity score. Varying from 1 to 20 the minimum number of matching peaks necessary to calculate a cosine score. For all following comparisons, min_match=10 was chosen. Please note that with increasing min_match parameter, more and more spectra pairs will not receive a Cosine score (bottom plot). For min_match=10, for instance, less than 10% of all spectra pairs will receive a Cosine score. For min_match=20 this drops below 5%. This also means potentially losing correctly matching pairs that just happen to not have sufficient shared peaks (unlike for Spec2Vec which would return a score for any pair).
