# Supplementary material for: Spec2Vec: Improved mass spectral similarity scoring through learning of structural relationships
Source: PLoS Comput Biol. 2021 Feb 16;17(2):e1008724. doi: 10.1371/journal.pcbi.1008724 (PMC7909622; doi:10.1371/journal.pcbi.1008724)
Supplement: S2 Text — (PDF) [file pcbi.1008724.s002.pdf]

Supporting Information S2 – Comparing different cosine score flavors

For main article "Spec2Vec: Improved mass spectral similarity scoring through learning of structural relationships", *Florian Huber, Lars Ridder, Stefan Verhoeven, Jurriaan H. Spaaks, Faruk Diblen, Simon Rogers, Justin J.J. van der Hooft.*

Comparing different cosine score flavors

Cosine scores come in many flavors, which makes it impossible to systematically compare all possible parameters settings and implementations. Apart from what we would consider the most basic implementation used here, we also tested cosine scores that weigh peaks according to their m/z ratio or their relative intensity. Settings that were tested in-depth stem from a suggestion from Demuth et al.[1] , as well as from current implementation used in NIST and Massbank [2]. Results across a wide range of min_match setting are shown in Figs B-D, while best case results for the different cosines score flavors are compared in Fig A.

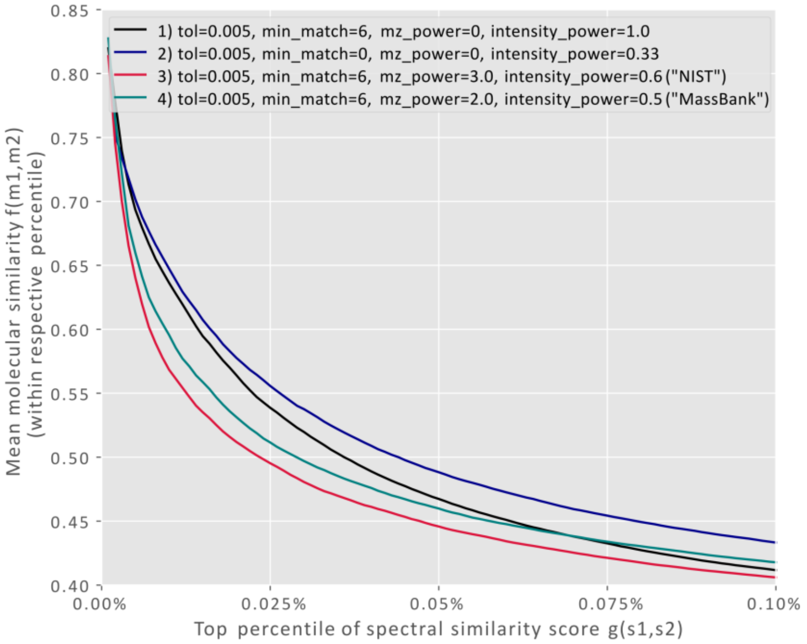

Fig A. Comparing different cosine score flavors. Four different mz_power/intensity_power parameter settings were tested. The plot shows the respective results with the best performing min_match criterion.

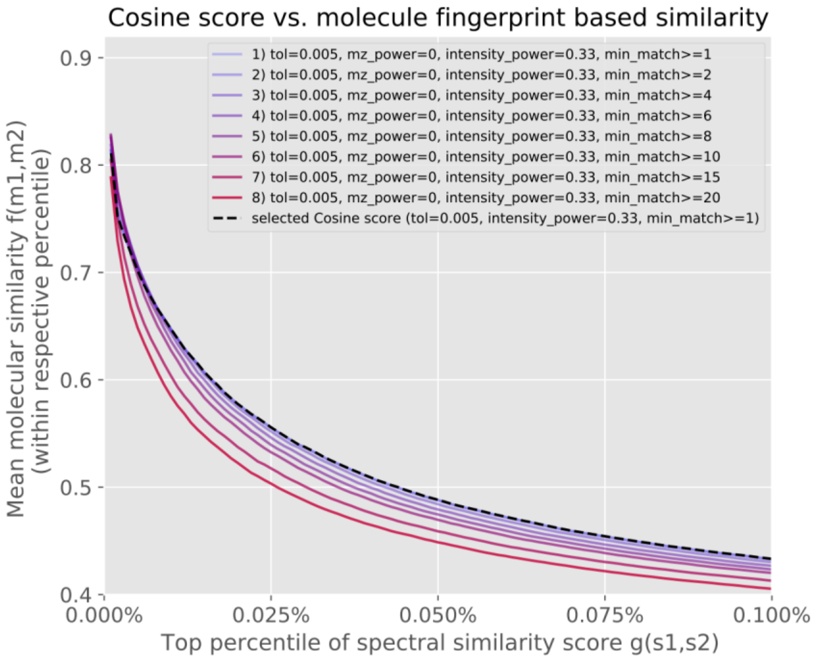

Fig B. Benchmarking of cosine score with intensity_power=0.33 (Demuth et al. [1]) for different min_match settings.

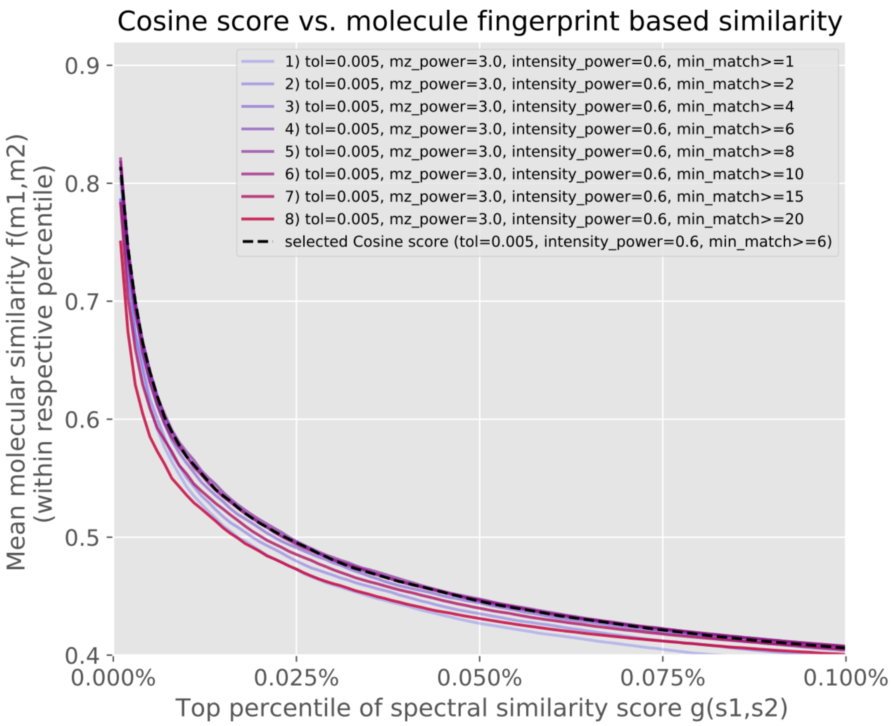

Fig C. Benchmarking of cosine score with mz_power=3.0 and intensity_power=0.6 (NIST settings [2]) for different min_match settings.

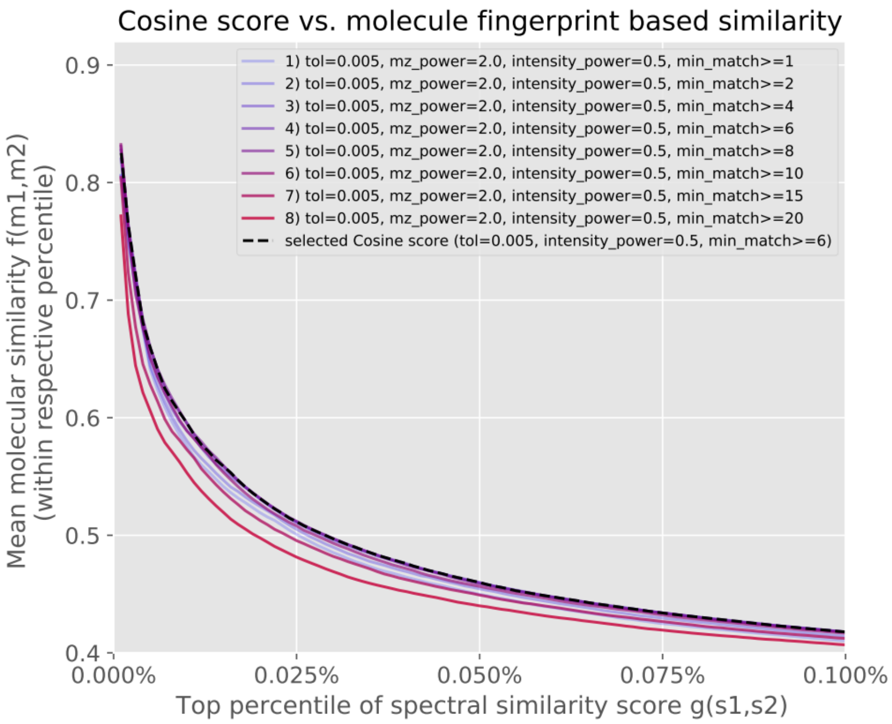

Fig D. Benchmarking of cosine score with mz_power=2.0 and intensity_power=0.5 (MassBank settings [2]) for different min_match settings.

References

1. Demuth W, Karlovits M, Varmuza K. Spectral similarity versus structural similarity: mass spectrometry. Anal Chim Acta. 2004;516: 75–85. doi:10.1016/j.aca.2004.04.014

2. Schollée JE, Schymanski EL, Stravs MA, Gulde R, Thomaidis NS, Hollender J. Similarity of High-Resolution Tandem Mass Spectrometry Spectra of Structurally Related Micropollutants and Transformation Products. J Am Soc Mass Spectrom. 2017;28: 2692–2704. doi:10.1021/jasms.8b05447
