# Supplementary material for: Spec2Vec: Improved mass spectral similarity scoring through learning of structural relationships
Source: PLoS Comput Biol. 2021 Feb 16;17(2):e1008724. doi: 10.1371/journal.pcbi.1008724 (PMC7909622; doi:10.1371/journal.pcbi.1008724)
Supplement: S3 Text — (PDF) [file pcbi.1008724.s003.pdf]

# Supporting Information S3 – Additional performance evaluations

For main article "Spec2Vec: Improved mass spectral similarity scoring through learning of structural relationships", *Florian Huber, Lars Ridder, Stefan Verhoeven, Jurriaan H. Spaaks, Faruk Diblen, Simon Rogers, Justin J.J. van der Hooft.*

Is this supplemental material we present additional evaluations of the modified cosine score reliability, and further inspect the computational performance of Spec2Vec (also in comparison to cosine-based scores). Finally we here further discuss the option to retrain pre-trained Spec2Vec model on additional data.

## Contents of supporting information S3

Analysis of modified cosine reliability ........ 2

Similarity score performance ........ 4

Model retraining ........ 8

References ........ 10

## Analysis of modified cosine reliability

We noted a large amount of false positive among the spectra pairs with high cosine or modified cosine scores. With false positives we here mean spectral pairs that receive a high spectra similarity but a low structural similarity score. This clearly shows in histograms of all structural similarities which belonged to pairs that received spectra similarity scores above a set threshold. For a given score, for instance modified cosine, we would select all pairs across the **UniqueInchikey** dataset (12,797 spectra, total of 81,875,206 unique pairs when excluding pairs of spectra with themselves) with a modified cosine score > 0.7, 0.8, etc. and create a histogram of the corresponding structural similarity values (Fig A). Even though we already use a relatively small peak m/z tolerance of 0.005 Da (preventing the collapse of too many mass fragments with different elemental formulas into one mass bin), the *min_match* criteria also clearly has to be raised to obtain acceptable results. In comparison, Spec2Vec similarities show a visibly lower amount of false positives (Fig A, lower right).

We also consider this one of the reasons for the notably poorer correlation between cosine and modified cosines scores and structural similarities (Fig 3 in main manuscript).

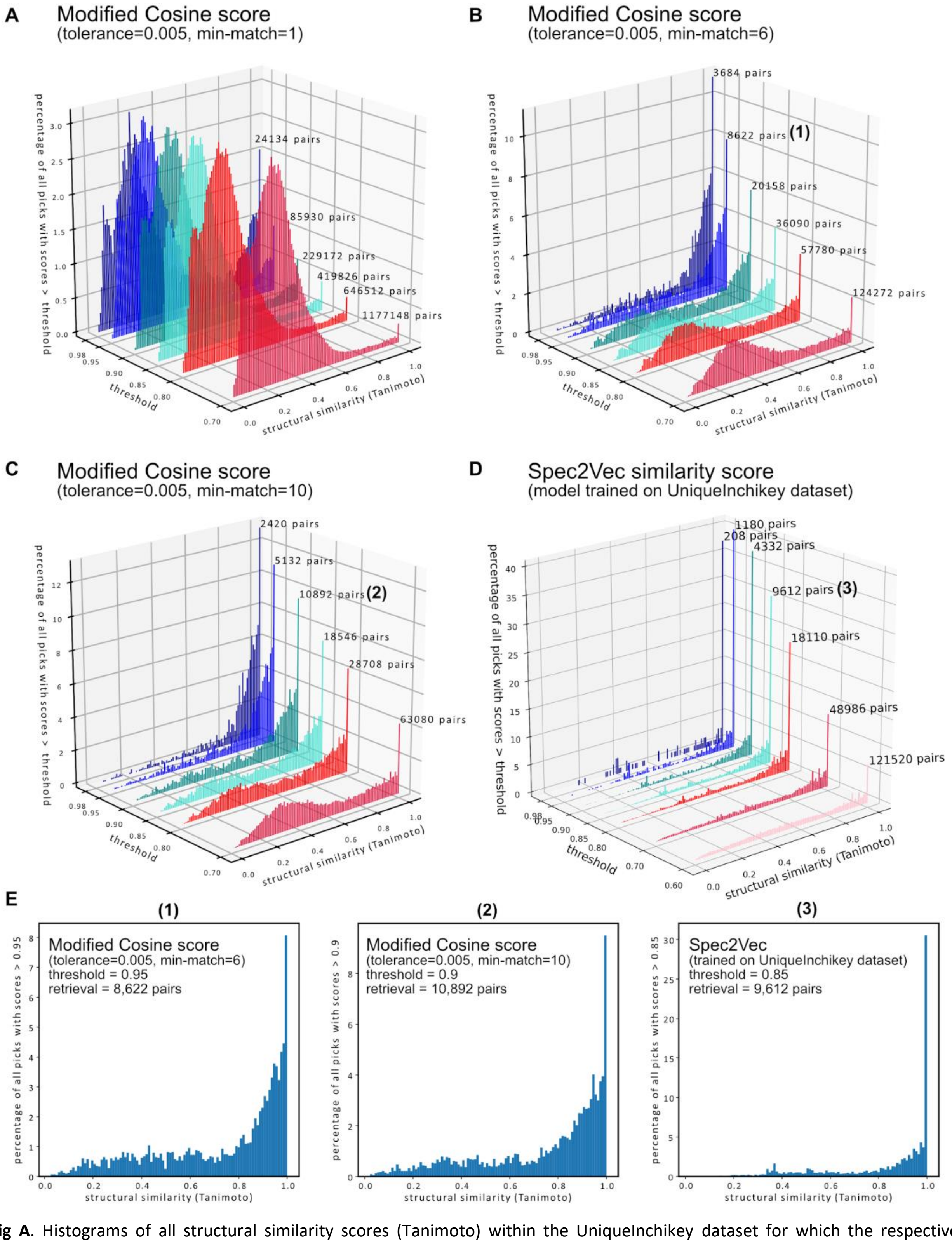


**Fig A**. Histograms of all structural similarity scores (Tanimoto) within the UniqueInchikey dataset for which the respective similarity scores are above a set threshold. Scores between identical spectra are excluded here. Thresholds between 0.7 to 0.98 were used to create the displayed histograms and are shown on the plot axis. The number of pairs that fitted the criteria (min-match and > threshold) is written on the top right of each histogram. (A) Modified cosine scores without requiring multiple machine peaks between two spectra results in drastic numbers of false positives (=low Tanimoto scores despite spectra similarity

scores > threshold). (B) Raising the minimum number of matches to 6 already improves the reliability of the modified cosine score notably. Still, even for comparably high thresholds (scores > 0.9 or > 0.95) a considerable fraction of all pairs with high modified cosine scores do not correspond to similar molecules. (C) For a minimum of 10 matching peaks the reliability further improved, but also the number of pairs fulfilling this criteria continues to drop. (D) Spec2Vec similarities also show notable levels of false positives, in particular for lower thresholds (0.7), but generally are visibly more shifted towards high Tanimoto scores when compared to the modified cosine scores. Absolute threshold values are hard to compare. For instance, a Spec2Vec similarity of 0.9 could be more or less common than a cosine score of 0.9 for a given setting. In (E) we hence compare three histograms that represent roughly equal numbers of spectra pairs (between 8,622 and 10,892). This shows that for comparable retrieval rates, high Spec2Vec scores correlate less frequently with very low molecular similarities.

## Similarity score performance

Jupyter notebook for performance analysis and plots: https://github.com/iomega/spec2vec_gnps_data_analysis/blob/master/notebooks/iomega-extra-classical-spectra-similarities-performance-analysis-synthetic-data.ipynb

When applying the different scores we noted large performance differences between the cosine-based score calculations and the Spec2Vec similarity calculations. Both the used implementation for the cosine scores (CosineGreedy) as well as Spec2Vec show computation times that scale linearly with the number of peaks in a spectrum (Fig B and E). An important difference is that Spec2Vec implementation is largely dominated by the step to compute the spectrum embeddings. When large arrays of spectra are compared, the implementation only computes the embeddings of all spectra once, and thereby increasingly reduces the problem to a simple cosine similarity calculation between arrays of 300-dimensional float vectors. This is a very efficient operation and results in a large performance advantage of Spec2Vec when compared to cosine-based scores whenever large arrays of spectra are compared, or -more generally- when the embeddings are calculated once and are stored (Fig D).

We also see such behavior when calculating very large all-vs-all similarity score matrices. For the UniqueInchikeys dataset, for instance, we computed the cosine-based similarity scores for all possible pairs between the 12,797 spectra (81,875,206 unique pairs), which --depending on the pre-processing and parameters used-- takes several hours on a standard CPU to compute. The precise computation time is highly dependent on the peak filtering, for the present noise filtering settings. When run on an Intel i7-8550U, the computation took 140 minutes (CosineGreedy). The number of matching peaks and the actual scores can be computed simultaneously (using matchms [1]) which allows to run the entire possible min_match range at once.

For comparison, calculating the Spec2Vec similarities for the same $81.8*10^6$ pairs took about 5 minutes (from spectra to scores, hence including the computationally more expensive embedding creation step).

Both implementations have not yet been optimized for parallel execution, which for such a task in principle is well suited. We expect that this could improve all mentioned scores (cosine-based and Spec2Vec) by a factor of 2 to 4 for the hardware used (Intel i7-8550U CPU, 4 cores).

In the following we present the results of some basic experiments for comparing performances of the different similarity scores. All results were computed on an Intel i7-8550U.

**Cosine score:**

At the heart of the cosine-based spectra similarity score between two spectra $S_1$ and $S_2$ lies a peak pairing or peak matching step which makes sure that each peak of $S_1$ cannot be matched to more than one peak of $S_2$. This represents an "assignment problem" which usually is approached by approximations to avoid that it takes polynomial time [2].

We now provide two implementations for deriving the cosine spectra similarity score within the Python package matchms. The implementation used for the present work is *CosineGreedy* which takes linear time due to the use of a greedy implementation. The underlying Python code code was further optimized using Numpy [3] and Numba [4]. The greedy implementation did make it possible to avoid the very undesired, sharp rise of computation time that can be seen for the exact solution which is provided as *CosineHungarian* (Hungarian algorithm using scipy [5], see Fig C).

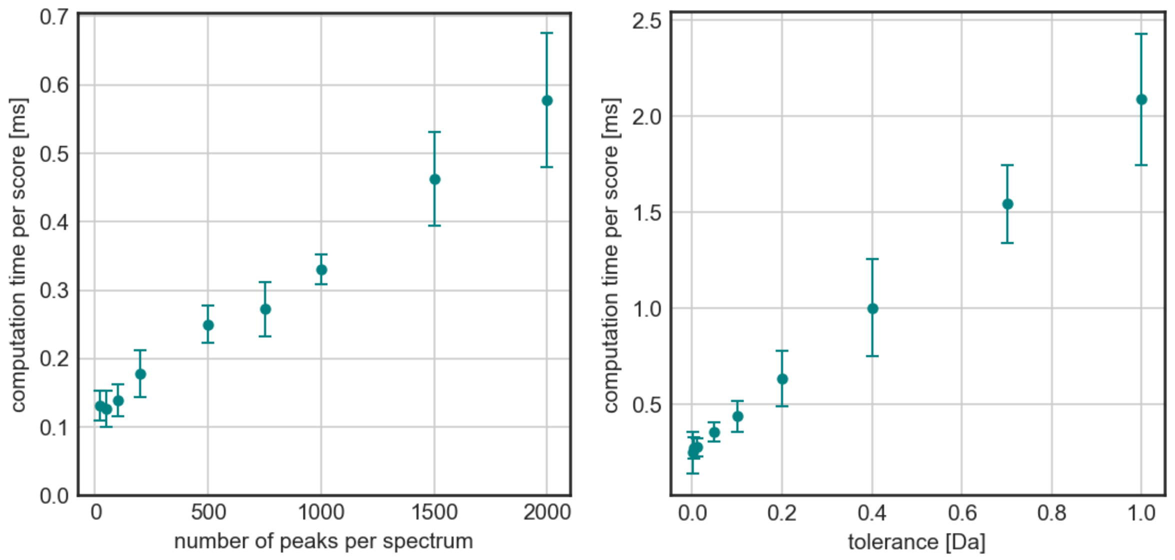


**Fig B**. (Left plot) Computation times for the cosine spectra similarity score as dependence of the number of peaks per spectrum. 100 spectra are randomly generated with the given number of peaks within the m/z range of [100.0, 600.0]. Used parameters were tolerance=0.005, mz_power=0, and intensity_power=1.0. For our CosineGreedy implementation the computation time takes linear time with respect to the number of peaks (as well as with the number of pairs to compute). The standard deviation over 10 independent runs is displayed as error bars. (Right plot) The underlying assignment problem is expected to scale with the number of pairs, which depends on the number of peaks, but also on the set tolerance value. Varying the tolerance from 0.001 Da to 1.0 Da for a randomly generated spectra (500 peaks within m/z range of [100.0 to 500.0]), the computation time increases linearly with the tolerance.

Hungarian implementation:

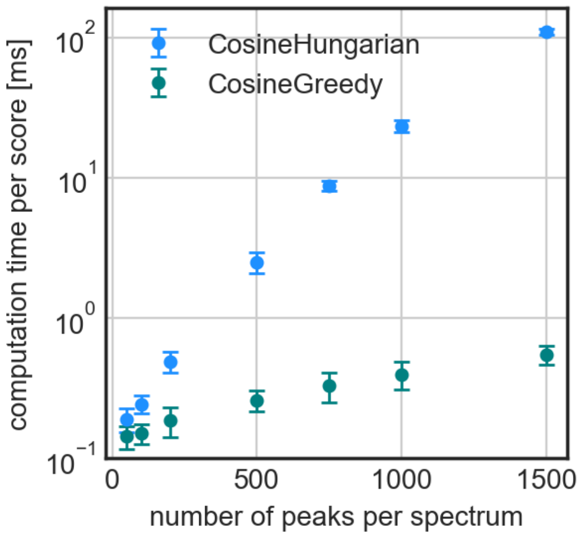


**Fig C**. Running the same experiment as the one shown in Fig B using the CosineHungarian implementation which is meant to achieve an exact solution of the underlying assignment problem. As expected, this implementation runs in polynomial time with drastically reduced performance for spectra that contain large numbers of peaks. We included the results for CosineGreedy for better comparison.

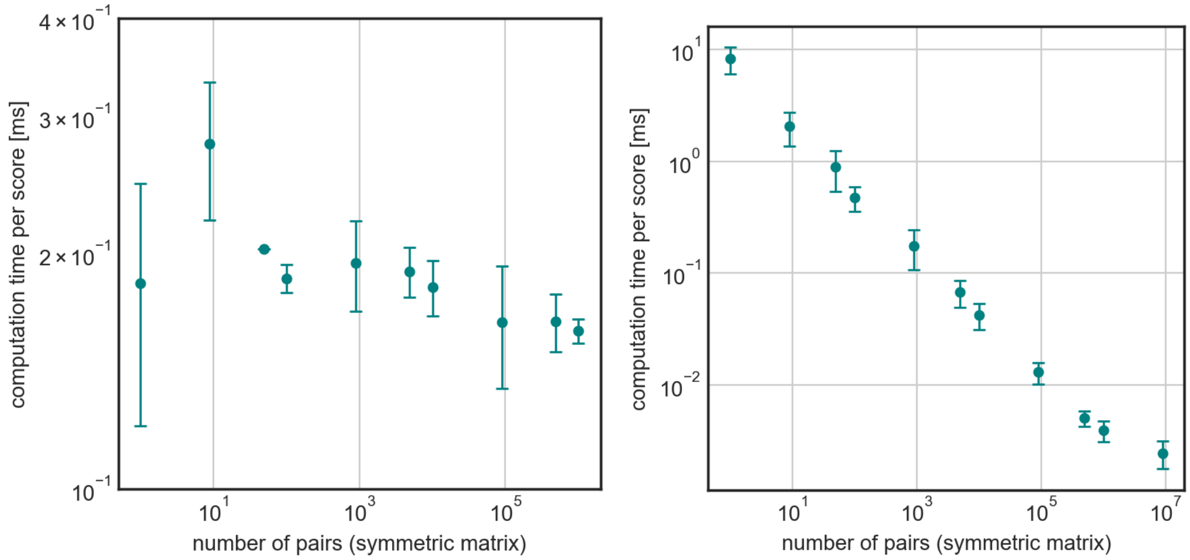


**Fig D**. Computing an all-vs-all CosineGreedy similarity matrix from N randomly generated spectra (200 peaks between 100.0 and 600.0 m/z), with N ranging from 1 to 1000, using CosineGreedy (left) and, with N ranging from 1 to 3000 using Spec2Vec (right). While the cosine spectra similarity calculation shows a constant computation time per score, Spec2Vec varies by several orders of magnitude when run on larger arrays of spectra simultaneously.

## Comparison to Spec2Vec similarity score

Deriving the Spec2Vec similarity between two spectra consists of three steps. The spectra need to be converted to documents (which is fast and was included in the preprocessing pipeline), then the documents are used to create an embedding vector, and finally the cosine similarity is calculated from the embedding vectors of two spectra.

Computationally, creating the embedding vector is the most expensive step. We hence provide a *Spec2Vec.matrix()* method that makes use of the fact that much fewer embedding needs to be calculated when comparing multiple spectra simultaneously. While computing a single similarity score between two spectra is generally slower than a cosine spectrum similarity score (if embeddings need be newly created), Spec2Vec is able to outperform the cosine spectrum similarity when larger arrays of spectra are compared to each other (see Fig E) or - more in general - when the spectra embeddings are created calculated once and then stored. The lower limit of this computation is the simple cosine similarity calculation between 300-dimensional float vectors which is a very fast and efficient operation ($6* 10^{-4}$ ms per score on an Intel i7-8550U CPU, see Fig F).

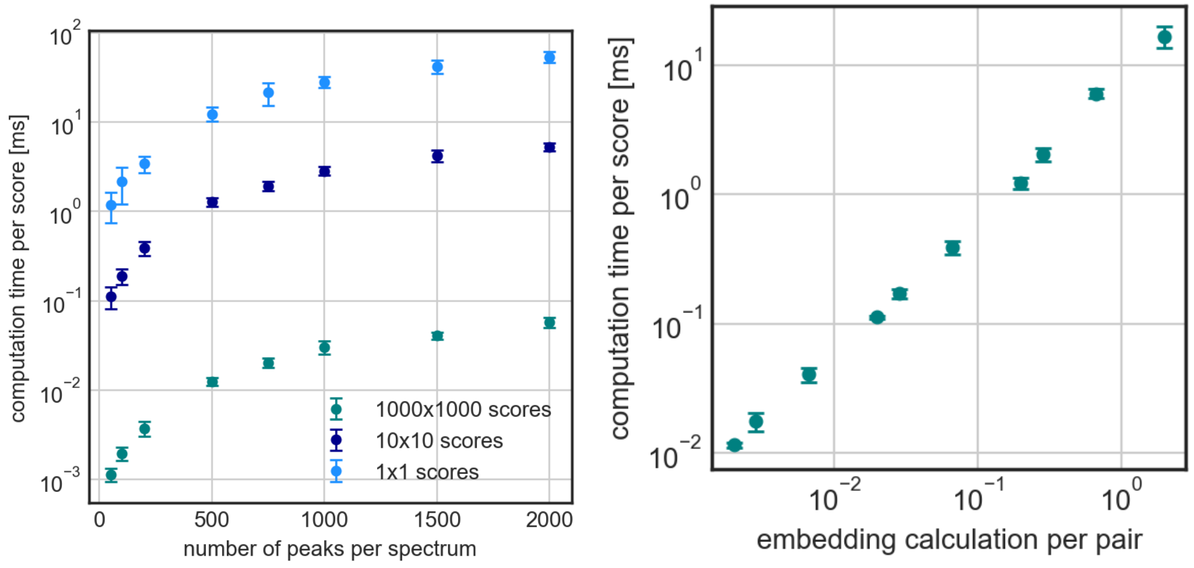


**Fig E.** (left plot) The computational cost for Spec2Vec similarities also increases linearly with the number of peaks in the spectra, but it drastically depends on the number of spectra that are compared simultaneously. (Right plot) Computing an all-vs-all similarity matrix from N randomly generated spectra (500 peaks between 100.0 and 600.0 m/z) using Spec2Vec, with N ranging from 1 to 1000. While N=1 does require 2 embeddings (reference + query spectrums) for 1 single score, N=1000 only requires deriving 2x1000 embeddings for $10^6$ scores. The lower limit here was estimated to be $6* 10^{-4}$ ms, which corresponds to the computational time of a cosine similarity calculation between two 300-dimensional float arrays (see Fig F).

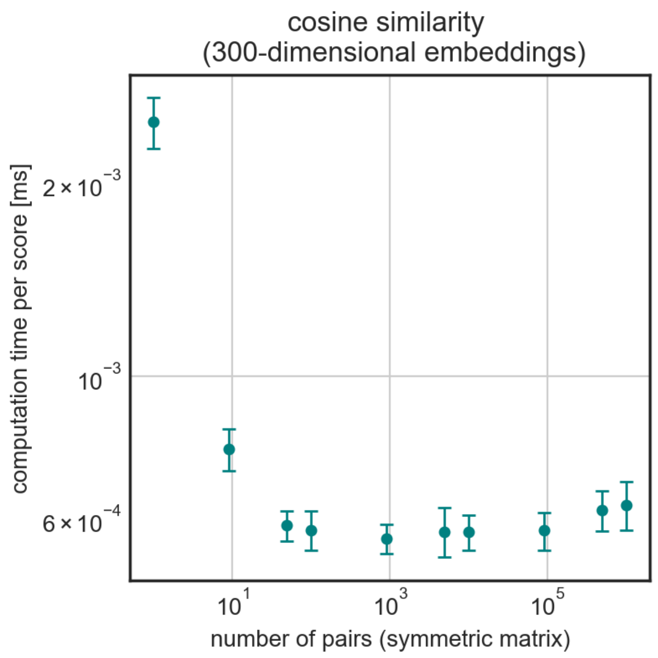


**Fig F.** Computing an all-vs-all similarity matrix from N randomly generated embedding vectors (300 dimensional). This can be considered the maximum achievable performance of Spec2Vec similarity calculations, for instance if spectrum embeddings are pre-calculated and stored.

## Model retraining

Jupyter notebook:
https://github.com/iomega/spec2vec_gnps_data_analysis/blob/master/notebooks/iomega-extra-evaluate-retraining-effect-5000subset.ipynb

Spec2Vec is an unsupervised technique. As such it allows it to be trained on the same data it later is applied on. In general, we see three main ways to apply Spec2Vec to a specific mass spectral dataset.

1) Using a pre-trained model (pre-trained on a large set of spectra, ideally with large overlap of instrument types and compound classes)
   We trained a model on a large set of positive ionmode spectra (**AllPositive** dataset), which can be downloaded here: https://doi.org/10.5281/zenodo.4173596
2) Updating a pre-trained model by a short additional training on the desired dataset.
3) Training a new model from scratch based on the desired set of spectra.

To explore the potential of updating an existing model by re-training it, we ran the library matching experiment (main article, Fig 4) and the unknown compound search experiment (main article, Fig 5) with additional re-training of the models using the query spectra. Interestingly, we

do not see any significant improvement for these cases and speculate that the data used for the initial training (>76,000 spectra) already contains enough features to cover the removed query spectra well enough. This is also in agreement with an additional experiment where different fractions of spectra were removed from the **UniqueInchikey** dataset (see Fig H).

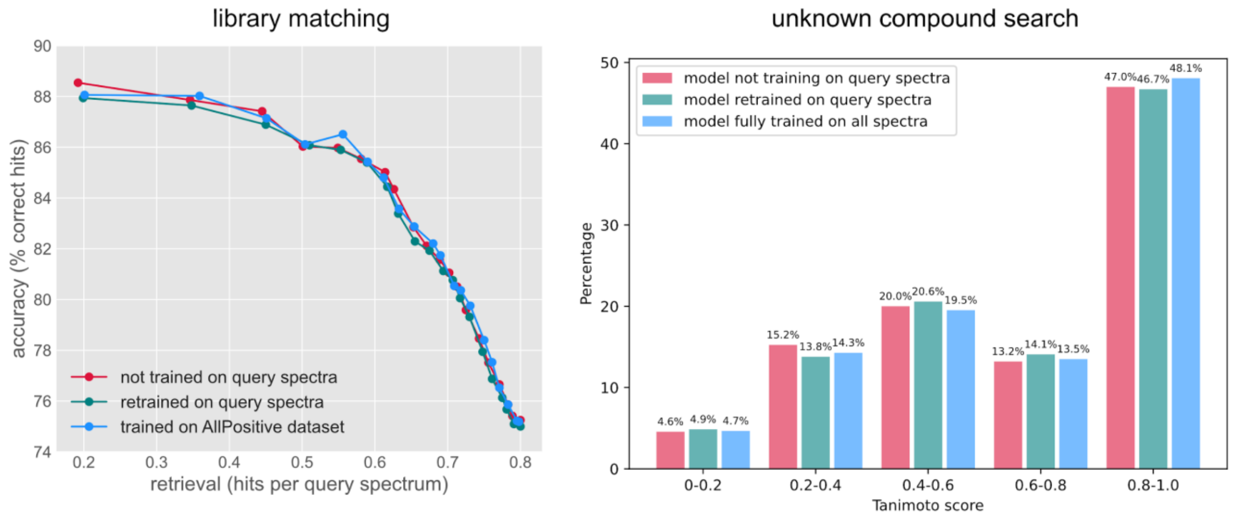


**Fig G.** (left plot) The library matching experiment (see main article, Fig 4) was also run using a Spec2Vec model that was retrained on the query spectral, and a Spec2Vec model trained on the entire **AllPositive** dataset. Differences between those three scenarios are very minor and can be expected to be within the error margin. (right plot) The same was done for the "unknown compound search" experiment (see main article, Fig 5), which again only shows a minor (or none) improvement when retraining the model or when including all query spectra in the initial training (training on entire **AllPositive** dataset).

In the experiment shown in Fig H it can be seen that models trained on only a small fraction of the data do strongly benefit from additional training on the missing data. However, for models trained on a large fraction of the data, the results of retraining usually remain inconclusive. We conclude that retraining is recommended in cases where a) the dataset Spec2Vec should be applied contains a significant number of spectra with respect to a pretrained model and/or b) if the training dataset contains a comparable small number of spectra. We would speculate that retraining might also be beneficial if the dataset in question is expected to contain spectra of notable different types (e.g. entirely different compound classes or instruments), though this would need further exploration.

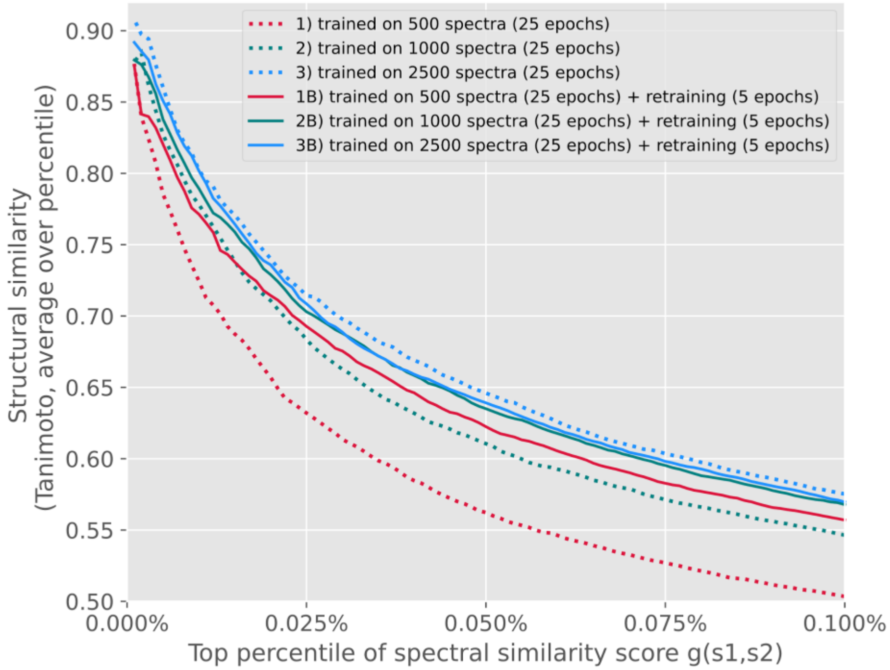


**Fig H.** Experiment on the effect of model retraining. Different models were tested on a subset of UniqueInchikey (containing 5000 spectra). Dotted lines: Models were trained on only a part of the dataset (500, 1000, or 2500 out of 5000 spectra) for 25 epochs using the before mentioned default settings. Solid lines: The same 3 models underwent retraining on the missing spectra for 5 epochs.

# References

1. Huber F, Verhoeven S, Meijer C, Spreeuw H, Castilla EMV, Geng C, et al. matchms - processing and similarity evaluation of mass spectrometry data. J Open Source Softw. 2020;5: 2411. doi:10.21105/joss.02411

2. Duan R, Pettie S. Linear-Time Approximation for Maximum Weight Matching. J ACM. 2014;61: 1:1–1:23. doi:10.1145/2529989

3. Harris CR, Millman KJ, van der Walt SJ, Gommers R, Virtanen P, Cournapeau D, et al. Array programming with NumPy. Nature. 2020;585: 357–362. doi:10.1038/s41586-020-2649-2

4. Lam SK, Pitrou A, Seibert S. Numba: a LLVM-based Python JIT compiler. Proceedings of the Second Workshop on the LLVM Compiler Infrastructure in HPC. Austin, Texas: Association for Computing Machinery; 2015. pp. 1–6. doi:10.1145/2833157.2833162

5. Virtanen P, Gommers R, Oliphant TE, Haberland M, Reddy T, Cournapeau D, et al. SciPy 1.0: fundamental algorithms for scientific computing in Python. Nat Methods. 2020;17: 261–272. doi:10.1038/s41592-019-0686-2
