# Supplementary material for: Spec2Vec: Improved mass spectral similarity scoring through learning of structural relationships
Source: PLoS Comput Biol. 2021 Feb 16;17(2):e1008724. doi: 10.1371/journal.pcbi.1008724 (PMC7909622; doi:10.1371/journal.pcbi.1008724)
Supplement: S4 Text — (PDF) [file pcbi.1008724.s004.pdf]

# Supporting Information S4 – Alternative scenario experiments

For main article "Spec2Vec: Improved mass spectral similarity scoring through learning of structural relationships", *Florian Huber, Lars Ridder, Stefan Verhoeven, Jurriaan H. Spaaks, Faruk Diblen, Simon Rogers, Justin J.J. van der Hooft.*

We here provide additional experiments to cover alternative scenarios for library matching or spectra network generation.

## Contents of supporting information S4


Library matching experiment - alternative scenario ........ 2

Unknown compound matching using modified cosine score ........ 3

Network analysis ........ 4

# Library matching experiment - alternative scenario

Jupyter notebook for alternative library matching scenario:
https://github.com/iomega/spec2vec_gnps_data_analysis/blob/master/notebooks/iomega-8-library-matching-scenario2.ipynb

For assessing the capacity of Spec2Vec to help find correct library matches we ran one scenario where all query spectra had at least one counterpart in the library data with a matching InChIKey (see Fig 4 from main article). In reality, however, it can be expected that many query spectra will not have a matching compound in the library. We here hence selected a different set of query spectra.

1500 randomly selected spectra were removed from a **AllPositive** such that we had: 500 spectra without a matching compound remaining, 500 spectra with exactly one spectrum each in the library of the same InChIKey and 500 spectra with > 1 remaining spectrum of same InChIKey (for the distribution of same-InChIKeys in the library, see inset in Fig A). Matching was done by pre-selecting spectra with the same precursor-m/z (tolerance = 1ppm) and then choosing the candidate with the highest spectral similarity score if this score was larger than a set threshold. Thresholds were then varied from 0.0 to 0.95, see Fig A).

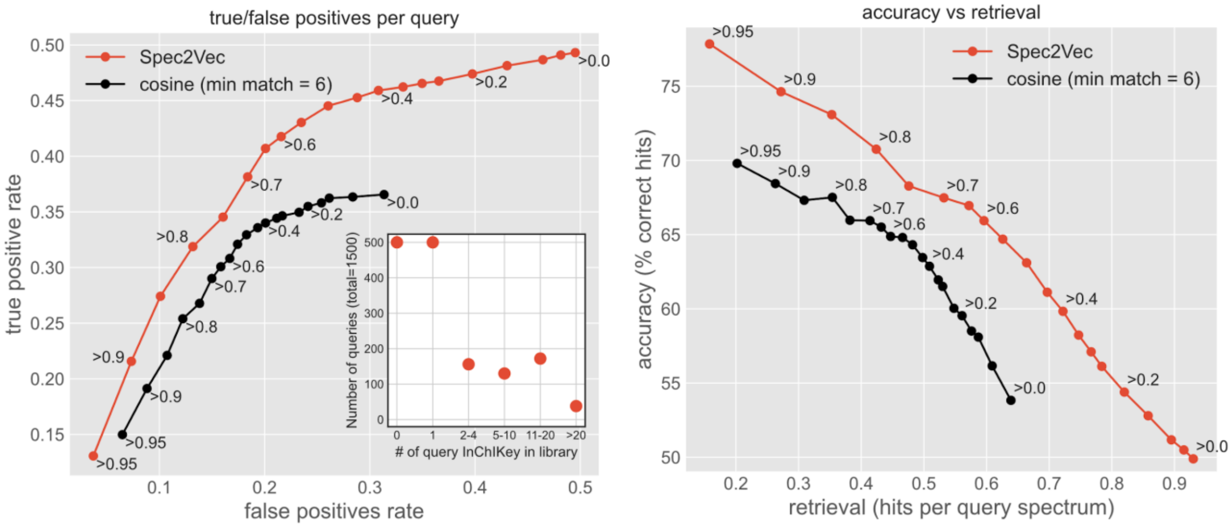


**Fig A.** Spec2Vec similarity scores deliver improved true-to-false-positive ratios during library matching. Here an scenario that differs from the one in the main manuscript in that it also contains query spectra for which there are no spectra in the library with matching InChIKey. The left plot shows the true-vs-false positive rate when using Spec2Vec (red) or cosine scores (black). Labels near the dots report the used similarity score thresholds. The inset plot on the left displays how many spectra identical InChIKey are part of the library for the 1500 query spectra. The plot on the right displays the resulting accuracy and retrieval rates for the same parameters. Using Spec2Vec, library matching could be done with notably higher accuracy across all tested retrieval rates.

As to be expected, the addition of spectra without matching compounds in the library resulted in a significantly higher false positive rate, as well as lower retrieval. As in the more optimistic case without unknown compounds, Spec2Vec displays higher accuracies and retrieval rates for

any given choice for the similarity score threshold. However, it also reveals that a reliable library matching procedure would likely benefit from further integrating additional measures such as fragmentation-tree-based methods to closely evaluate the selected candidates and reduce the number of false positives.

## Unknown compound matching using modified cosine score

Jupyter notebook:
https://github.com/iomega/spec2vec_gnps_data_analysis/blob/master/notebooks/iomega-9-unknown-compound-search-mod-cos.ipynb

Analogous to the experiment on matching spectra of compounds which are absent from the library data (see Fig 5 in the main article), we also ran the same analysis using the modified cosine score instead of Spec2Vec. Parameters used were min_match=6 and tolerance=0.005. Finding the highest 10 modified cosine scores out of 76,062 library spectra for a total of 1,030 query spectra took 165 minutes on an Intel i7-8550U CPU. This was significantly slower than the same routine run with Spec2Vec similarities (2.5 minutes), but can still be considered feasible for such a task.

Interestingly, the modified cosine score shows a very similar tendency to select structurally similar compounds with much higher accuracy for larger compounds (Fig B-B). Overall, the library-based suggestions based on the top-10 Spec2Vec scores represented more reliable suggestions (Fig B-A). However, the aim of this matching experiment was not to fully benchmark the performance of those two scores against each other. We rather believe that the displayed accuracies should be interpreted as lower bound of what these two scores can do. Results can almost certainly be improved by careful parameter optimization and including minimum score threshold. Even more interesting will be to further explore the complementary nature of these two different similarity measures. From first, very naive, tests on combining both scores (see Fig C), we would expect that a combined approach could greatly reduce the fractions of false suggestions (Tanimoto < 0.6 in 2A).

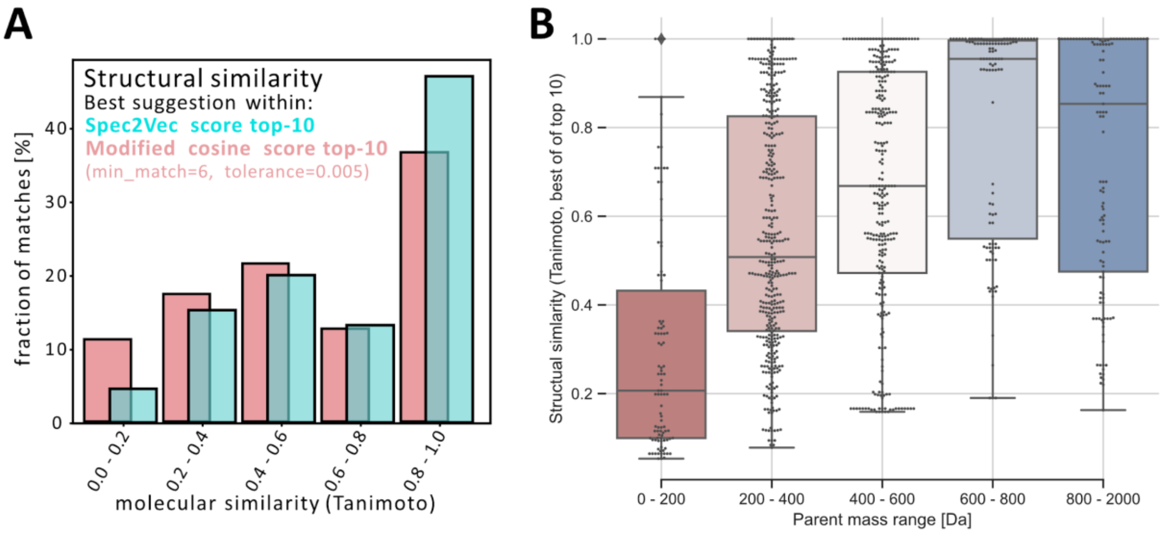


**Fig B.** 1030 query spectra were compared to 76,062 library spectra, none of which had a matching InChIKey (same experiment as in Fig 5 in the main article). (A) Comparing the best Tanimoto score within the highest 10 similarities found by either Spec2Vec or modified cosine score. Both methods correctly suggest structurally related compounds (Tanimoto > 0.6), but Spec2Vec overall gives more reliable suggestions. (B) Analogous plot to Fig 5B, main article when run with modified cosine score instead of Spec2Vec, showing the dependency of the suggested matches with the query compound’s parent mass.

## Network analysis

Based on the different working principles of cosine-based spectral similarity scores, and Spec2Vec similarity, we expect different weaknesses and strengths of both score types. Those differences could be exploited by combining scores. In a first, and highly simplified, test on the described molecular networking task (see main article, Fig 6), we observed that a simple linear combination of both modified cosine and Spec2Vec similarities only mildly lowers the total fraction of clustered spectra, but notably reduces fraction of poorly clustered spectra (Fig C). We can hence increase clustering accuracy by relying on both scores, which seems a promising starting point to build upon in future work.

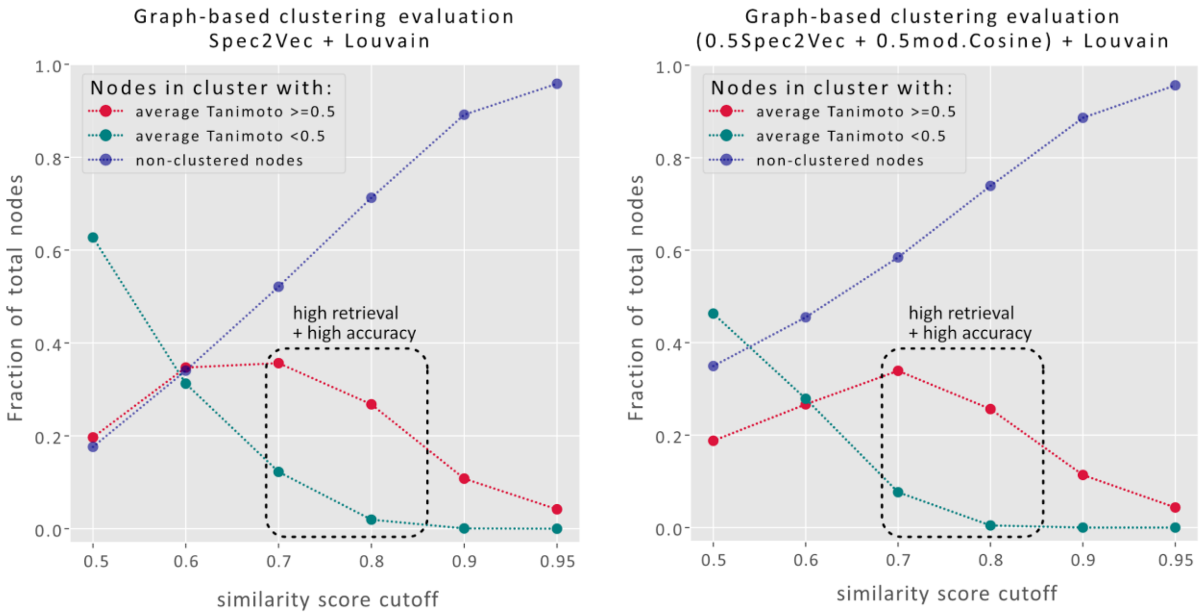


**Fig C.** Networks were generated from spectra (nodes) by adding links based on spectra similarities, as shown in Fig 6 (main article). The left plot is identical to the right plot in Fig 6 (main manuscript) and was generated by only using Spec2Vec similarity scores. On the right we used a simple combination of Spec2Vec similarities and modified Cosine scores (similarity = 0.5 Spec2Vec similarity + 0.5 mod.Cosine, min_match=10, tolerance=0.005). Already this simple combination leads to a drop of the loss structural similarity clusters (green) with respect to the well-clustered fraction (red), effectively leading to a higher accuracy. We expect that more refined combinations of both similarity scores can be used to further build upon these results.
