# Supplementary material for: Spec2Vec: Improved mass spectral similarity scoring through learning of structural relationships
Source: PLoS Comput Biol. 2021 Feb 16;17(2):e1008724. doi: 10.1371/journal.pcbi.1008724 (PMC7909622; doi:10.1371/journal.pcbi.1008724)
Supplement: S5 Text — (PDF) [file pcbi.1008724.s005.pdf]

Supporting Information S5 – Spec2Vec in GNPS environment

For main article "Spec2Vec: Improved mass spectral similarity scoring through learning of structural relationships", *Florian Huber, Lars Ridder, Stefan Verhoeven, Jurriaan H. Spaaks, Faruk Diblen, Simon Rogers, Justin J.J. van der Hooft.*

Spec2Vec in GNPS environment

To start making Spec2Vec available within the GNPS [1] environment (https://gnps.ucsd.edu), an option to calculate Spec2Vec scores of positive ionisation mode classical and feature-based molecular networks has been added to GNPS release 27. There is a link "Network with Spec2vec" that reads in the spectral mgf file and graphml file of the molecular networking job and from which you can start the Spec2Vec analysis. The default GNPS-Spec2Vec analysis will take the spectral data from the molecular network and use a pre-trained model to calculate Spec2Vec similarity scores. Subsequently, all cosine pairs and Spec2Vec pairs below a user-provided threshold score (0.7 by default) are deleted prior to reconstructing the Spec2Vec-based spectral network (with by default a topK of 100 - a parameter that breaks up the larger molecular families to prevent hairball formation).

The resulting Spec2Vec-based spectral network can be visualized directly in the browser - or on your local computer by downloading the graphml file. In the future, we envision to also include the option to calculate negative ionisation mode data, library matching based on Spec2Vec scores, among other ideas.

Here below are links to positive ionisation mode examples from the MolNetEnhancer [2] molecular networking jobs and the example feature-based molecular networking data from GNPS:

Feature-based Molecular Networking

GNPS FBMN example data (subset of American Gut Project samples of volunteers consuming higher and lower amounts of vegetables and fruit)

- Molecular Networking job release V27:
  https://gnps.ucsd.edu/ProteoSAFe/status.jsp?task=470836933ab047628f11f903af873b0a
- GNPS-Spec2Vec analysis:
  https://gnps.ucsd.edu/ProteoSAFe/status.jsp?task=b4b582fdd4844adeaf601bf76cc0d72f

Euphorbia dataset:

- Molecular Networking job release V27:

https://gnps.ucsd.edu/ProteoSAFe/status.jsp?task=a3ca5a96f62b4a0a9043a9d2bb9625bb

- GNPS-Spec2Vec analysis: https://gnps.ucsd.edu/ProteoSAFe/status.jsp?task=b2cb48a96be84efb86a459612546ff01

Classical Molecular Networking

Streptomyces and *Salinispora* dataset:

- Molecular Networking job release V27: https://gnps.ucsd.edu/ProteoSAFe/status.jsp?task=8e98a739c59a45689f2cb891418402c4
- GNPS-Spec2Vec analysis: https://gnps.ucsd.edu/ProteoSAFe/status.jsp?task=62361a03cb634f86ad5eacf55834d1cc

Photorhabdus and *Xenorhabdus* dataset:

- Molecular Networking job release V27: https://gnps.ucsd.edu/ProteoSAFe/status.jsp?task=e9e8edc75ae84bbb9034d8ab5ec956ec
- GNPS-Spec2Vec analysis: https://gnps.ucsd.edu/ProteoSAFe/status.jsp?task=fa96df30b52b4824ad6076bc52ca358a

References

1. Wang M, Carver JJ, Phelan VV, Sanchez LM, Garg N, Peng Y, et al. Sharing and community curation of mass spectrometry data with GNPS. Nat Biotechnol. 2016;34: 828–837. doi:10.1038/nbt.3597

2. Ernst M, Kang KB, Caraballo-Rodríguez AM, Nothias L-F, Wandy J, Chen C, et al. MolNetEnhancer: Enhanced Molecular Networks by Integrating Metabolome Mining and Annotation Tools. Metabolites. 2019;9: 144. doi:10.3390/metabo9070144
